# Retinoic Acid Informs the Positional Identity of Frontonasal Neural Crest Cells Through Alx Family of Transcription Factors

**DOI:** 10.1096/fj.202601339R

**Published:** 2026-06-09

**Authors:** Shuxuan Wu, Yifan Lu, Yixin Tu, Yuke Xi, Qianxue Wan, Minghui Yue, Huan Liu, Zhaoming Wu

**Affiliations:** ^1^ Division of Applied Oral Sciences and Community Dental Care, Faculty of Dentistry The University of Hong Kong Hong Kong China; ^2^ Affiliated Stomatological Hospital of Xuzhou Medical University, School of Stomatology Xuzhou Medical University Xuzhou China; ^3^ State Key Laboratory of Oral & Maxillofacial Reconstruction and Regeneration, Key Laboratory of Oral Biomedicine of Ministry of Education, Hubei Key Laboratory of Stomatology, School & Hospital of Stomatology Wuhan University Wuhan China; ^4^ Frontier Science Center for Immunology and Metabolism Wuhan University Wuhan China; ^5^ TaiKang Center for Life and Medical Sciences Wuhan University Wuhan China

**Keywords:** *Alx1*, chick, cranial neural crest cells, facial cleft, frontonasal development, retinoic acid signaling

## Abstract

Cranial neural crest cells (CNCCs) give rise to the majority of the skeletal elements of the face. The precise morphogenesis of the face relies on highly coordinated actions of CNCCs, requiring each CNCC to obtain correct positional identity. As a diffusive signaling molecule, retinoic acid (RA) is known to regulate the positional identities along the anterior–posterior axis of the developing hindbrain by activating the Hox family of transcription factors. However, whether RA also has a direct role in regulating the positional identities of Hox‐negative CNCCs, which give rise to the skeletal framework of the face, was unclear. In this study, we show that RA acts as a local environmental cue that patterns the Hox‐negative CNCCs by activating the Alx family of transcription factors. We observed midfacial dysplasia and midline facial clefting in chick embryos after blocking RA signaling with an inverse pan‐RAR agonist. Gene expression analysis revealed that this morphological defect is associated with the transformation of frontonasal neural crest identity toward a first pharyngeal arch (PA1)‐like identity, a patterning defect that was also observed in *Alx1* and *Alx4* compound mutant mouse embryos. We further showed that both *Alx1* and *Alx4* are regulated by RA through cell‐autonomous RA receptor (RAR) signaling. Mechanistically, RA signaling regulates *Alx1* through an evolutionarily conserved distal enhancer located upstream of *Alx1* within the intronic region of the gene *Lrriq1*, whereas the RA‐responsiveness of *Alx4* is conferred by its promoter. These findings establish a mechanistic linkage between RA signaling and Alx genes and provide novel insights into the craniofacial defects associated with disrupted RA signaling.

## Graphical abstract

Retinoic acid (RA) regulates the positional identity of cranial neural crest cells (CNCCs) through the transcription factors *Alx1* and *Alx4*. Upon receiving RA from the local environment, liganded RAR/RXR heterodimers activate the expression of *Alx1* and *Alx4*, which promote the frontonasal identity of CNCCs and inhibit the first pharyngeal arch identity of CNCCs.

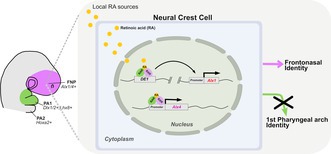

## 1 Introduction

Cranial neural crest cells (CNCCs) are the building blocks of the facial skeletal elements. CNCCs originate from the neural plate border where they delaminate and migrate along the dorsal‐ventral axis to populate the mesenchyme of the facial primordia, comprising the frontonasal prominence (FNP) and the paired first pharyngeal arch (PA1). The FNP is subsequently divided by the nasal placodes into the paired lateral nasal processes (LNP) and median nasal processes (MNP), while the PA1 gives rise to the paired maxillary processes (MxP) and mandibular processes (MdP). During facial morphogenesis, these facial prominences undergo coordinated outgrowth and fusion, with the FNP contributing to the forehead and nasal structures and PA1 forming the upper and lower jaws [1].

After populating the facial primordia, CNCCs express specific combinations of positional genes according to their locations in the embryo, forming a blueprint for the future steps of tissue morphogenesis and cell differentiation. How CNCCs obtain their positional identity has long been an important question in craniofacial development. Whereas the expression of Hox genes seems largely dependent on the origin of pre‐migratory CNCCs along the rostral‐caudal axis [2], the further regionalization of Hox‐free CNCCs in different facial primordia appears to rely mainly on local environmental cues [3, 4].

Midfacial development is closely associated with a group of *aristaless‐like* homeobox (Alx) transcription factors, including *Alx1*, *Alx3*, and *Alx4* in mammals [5]. Mutations in Alx genes in humans cause frontonasal dysplasia (FND), a congenital condition that is characterized by midfacial developmental defects [6]. Notably, *ALX1* mutations in humans cause FND3, the most severe form of FND [7]. Studies in zebrafish and patient‐derived iPSCs suggested that *Alx1* plays a role in neural crest migration [8, 9, 10]. A mouse genetics study demonstrated that Alx genes are required for establishing the positional identity of CNCCs in the FNP, evidenced by the expression of ectopic PA1 NCC identity marker genes in the developing FNP of *Alx1/Alx4* compound mutant mouse embryos [11].

Retinoic acid (RA), the active metabolic derivative of vitamin A, functions as a diffusive signaling molecule and is essential for embryonic development [12]. RA binds to nuclear retinoic acid receptors (RARs)‐RARα, RARβ and RARγ. These receptors form heterodimers with retinoid X receptors (RXRs) and function as ligand‐dependent transcription factors. The RAR/RXR complex, when activated by RA, can bind to RA response elements (RAREs) to regulate the transcription of its target genes [12]. RA signaling is necessary for the development of FNP‐derived structures; both deficiency and excess in RA signaling in the developing FNP during early embryogenesis result in a variety of craniofacial deformities [13, 14, 15, 16]. While RA is known to regulate facial morphogenesis through moderating signaling exchange between CNCCs and facial ectoderm or neuroepithelium [16, 17], the cell‐autonomous role of RA‐activated RAR signaling within CNCCs is less understood, despite its importance underscored by the severe craniofacial defects caused by neural crest cells (NCCs)‐specific ablation of RARα/RARβ/RARγ in mouse embryos [18].

In this study, we investigated the roles of RA signaling in patterning the regional identities of CNCCs. We demonstrated that RA signaling acts as a local environmental cue to inform the positional identities of CNCCs in the FNP through promoting the expression of the Alx family of transcription factors. Importantly, our data showed that the Alx‐dependent establishment of the frontonasal identity in CNCCs within the FNP relies on cell‐autonomous functions of RARs. Analyses of *Alx1* and *Alx4 cis*‐regulatory elements revealed that they are likely direct downstream targets and effectors of RA signaling during CNCCs patterning.

## 2 Materials and Methods

### 2.1 Chick Embryos and Beads Implantation

Fertile chicken eggs were incubated at 38°C and staged according to the Hamburger and Hamilton staging system [19]. Resin exchange beads (Sigma, USA) were soaked in 5 mg/mL BMS493 (STEMCELL Technologies, Canada) dissolved in dimethyl sulfoxide (DMSO; Sigma, USA) for 30 min at room temperature (RT), as previously described [20]. Control beads were soaked in DMSO. The beads were then rinsed in PBS containing 0.1% Fast Green for visualization and implanted into the rostral end of the forebrain of HH9‐10 chick embryos. After the procedure, the eggs were resealed and incubated until the embryos reached the desired stages. All animal experimental procedures were approved by the Committee on the Use of Live Animals in Teaching and Research (CULATR) of the University of Hong Kong and conformed to the updated ARRIVE 2.0 (Animal Research: Reporting of In Vivo Experiments) guidelines.

### 2.2 Facial Explant Culture

The heads of HH17‐18 chick embryos were bisected through the midline, and each half was cultured on a filter membrane at the liquid‐air interface for 24 h. The lower culture medium consisted of Dulbecco's Modified Eagle Medium (DMEM, Gibco, USA) supplemented with 10% fetal bovine serum (FBS, Gibco, USA), penicillin (100 IU/mL, Gibco, USA), and 1% GlutaMAX (Gibco, USA).

### 2.3 *In Ovo* Electroporation

*In ovo* electroporation was carried out as previously described [21]. pCIG‐RARa‐403‐myc‐GFP was a gift from Prof. Thomas Jessell (Addgene plasmid #16286) [22]. Briefly, DNA solutions at a concentration of 2 μg/mL containing 0.05% Fast Green were injected into the developing neural tube of HH8 chick embryos using a glass capillary. The electrodes were positioned on the vitelline membrane on either side of the neural tube. Four square wave pulses, each with an amplitude of 32 V, a duration of 50 ms, were applied to the embryo with an inter‐pulse interval of 950 ms. After electroporation, the embryos were resealed and incubated to the desired stage.

### 2.4 Preparation of Embryos

Chick embryos were harvested, dissected in phosphate‐buffered saline (PBS), and collected in tubes on ice. Dissected embryos were washed in PBS and fixed in 4% paraformaldehyde overnight at 4°C. Chick embryos were washed three times in PBS/0.1% Tween20 (PBST). They were dehydrated in methanol (MeOH)–PBST bath series: 25% MeOH/75% PBST, 50% MeOH/50% PBST, 75% MeOH/25% PBST, and 100% MeOH. The embryos were stored in MeOH at −20°C for later use.

### 2.5 Histology and Skeletal Staining

For histological analyses, chick embryos were dissected at desired stages, fixed in 4% paraformaldehyde, dehydrated through an ethanol series, embedded in paraffin, sectioned at 7 μm thickness, and stained with Alcian blue followed by hematoxylin and eosin. For skeletal staining, chick embryos were fixed at stage 38 (after 11–12 days of incubation) using 100% ethanol for 4 days. Next, embryos were stained with Alcian Blue (0.3% Alcian Blue in acetic acid and 95% ethanol) for 3 days. After washing in 95% ethanol, soft tissues were dissolved in 2% KOH overnight and stained with Alizarin Red (50 μg/mL in 1% KOH) for 24 h. Chick embryos were then cleared in 20% glycerol/1% KOH solution, photographed, and stored in 50% glycerol/50% ethanol solution.

### 2.6 Whole‐Mount Hybridization Chain Reaction (HCR)

All DNA oligo probes were synthesized by Integrated DNA Technologies Company (IDT, Singapore). The probe sequences are listed in Table S1. The HCR procedure was based on a previous report [23] with minor adjustments. Briefly, chick embryos stored in MeOH were rehydrated through a MeOH/PBST gradient series. Embryos were permeabilized with 10 µg/mL proteinase K and post‐fixed in 4% PFA for 15 min at RT. Subsequently, embryos were washed three times in PBST, followed by two washes in 5 × SSCT. After pre‐hybridizing in 500 μL hybridization buffer (HCR Buffers, Molecular Instruments, USA) for 30 min at 37°C, the embryos were incubated overnight at 37°C in hybridization solution containing probes targeting genes (4 pmol in 500 μL hybridization buffer). After hybridization, samples were washed four times using HCR washing buffer (HCR Buffers, Molecular Instruments, USA) at 37°C, followed by two washes in 5xSSCT at RT. Next, after 5 min of pre‐amplification in 500 μL amplification buffer (HCR Buffers, Molecular Instruments, USA), the embryos were kept in amplification solution overnight in the dark at RT. After amplification, embryos were washed in 5xSSCT three times at RT. Embryos were imaged under a fluorescent stereo microscope.

### 2.7 Cryosection and Quantification

Following HCR, embryos were fixed in 4% PFA overnight at 4°C, dehydrated through a graded sucrose series (10%, 20%, and 30%), and equilibrated in a 1:1 mixture of 30% sucrose and OCT compound (Sakura Finetek, Japan). Embryos were then embedded in OCT in cryomolds, frozen in liquid nitrogen, and stored at −20°C before cryosectioning. Cryosections were prepared using a cryostat (Leica, USA), mounted on Superfrost Plus slides (Epredia, USA), and stored at −20°C. Before imaging, slides were washed in PBST to remove OCT, counterstained with DAPI (Invitrogen, USA), and mounted in FluorSave Reagent (Sigma, USA). Sections were imaged and analyzed using a confocal microscope. For quantification, three sections from each embryo were analyzed, and the average number of HCR‐positive dots per cell was quantified in both GFP+ and GFP− cells. Data were processed using GraphPad Prism 9.0 and are presented as mean ± SEM. Statistical analyses were performed using two‐way ANOVA or an unpaired *t*‐test. A *p*‐value < 0.05 was considered statistically significant.

### 2.8 Whole‐Mount In Situ Hybridization (ISH)

Whole‐mount in situ hybridization was performed as previously described [11]. The RNA probes were designed based on their mRNA sequences. The plasmid templates for *Alx1 and Alx4* probes were amplified by PCR and cloned into the PCRII vector using the following primers: c*Alx1*‐F: 5′‐AGGCACTTTGGAGCACGTTA‐3′, c*Alx1*‐R: 5′‐AATCTGTCCGGGGTGAATGG‐3′; c*Alx4*‐F: 5′‐GCAGCACTTTGAGCTACCCT‐3′, c*Alx4*‐R: 5′‐TTCTGCAAGGTGGCAGTAGG‐3′. RNA probe was synthesized through in vitro transcription and purified through LiCl RNA precipitation.

### 2.9 Generation of the Alx1 Expression Construct

The coding sequence of the chick *Alx1* gene was amplified using Q5 polymerase (NEB, USA) from a chick embryo cDNA library. The amplified gene fragment was inserted into the multiple cloning site of the pCIG‐GFP expression vector to generate the pCIG‐Alx1‐GFP construct.

### 2.10 Transfection and Luciferase Assay

The putative sequences of mouse *Alx1* promoter (*Alx1promo*), *Alx1* enhancer (*Alx1‐DE1*), *Alx3* enhancer (*Alx3‐DE*) and *Alx4* promoter (*Alx4promo*) were amplified by PCR and cloned into the *p*GL3‐basic vector to build the firefly luciferase reporters. The primers for PCR were: *Alx1promo‐F*: 5′‐GAAATGTGGTGAACCGGGTG‐3′, *Alx1promo‐R*: 5′‐TGCAGTGGCATCTAACGGAAT‐3′; *Alx1‐DE1‐F*: 5′‐TCCTCAGTCTTCACAGATACGC‐3′, *Alx1‐DE1‐R*: 5′‐TGAGCAATAAAATGGCGGTGC‐3′; *Alx3‐DE‐F*: 5′‐GAATATGCCTATGCCGTGGG‐3′, *Alx3‐DE‐R*: 5′‐AACTTGACAGAGTGTGGTGACA‐3′; *Alx4promo‐F*: 5′‐CCAACGGGTTGACAGAGCTA‐3′, *Alx4promo‐R*: 5′‐GGACAGACCCGGAGTTCCTC‐3′. *p*cDNA3.1‐hRARα (Addgene plasmid #135397) and *p*cDNA3.1‐hRXRα (Addgene plasmid #135910) were gifts from Prof. Catharine Ross [24]. HEK293T cells were transfected with the luciferase reporter constructs, *p*RL‐TK containing the *Renilla* luciferase gene, *p*cDNA3.1‐hRARα and *p*cDNA3.1‐hRXRα plasmids via Lipofectamine 3000 reagents (Invitrogen, USA). Twenty hours after the transfection, the cells were treated with 0.5 μM of all*‐trans* RA or DMSO. Six hours after the treatment, luciferase activities were assessed using a Dual‐Luciferase Reporter Assay System (Promega, USA) following the manufacturer's protocol. Transfections were performed in triplicate, and each experiment was repeated at least three times. Results were presented as the ratio of firefly to Renilla luciferase activity (means ± SEM). Statistical significance was determined using two‐way ANOVA. *p*‐values < 0.05 were considered statistically significant in all experiments.

### 2.11 Bioinformatics Analysis

The signal track of E10.5 FNP ATAC‐seq (GSE89436), ChIP‐seq peaks of zebrafish RARαa ChIP‐seq (GSE233695), and PCHi‐C results from E10.5 FNP (GSE211902) (*mm10*) were downloaded from the Gene Expression Omnibus (GEO). The BED files of ChIP‐seq peaks of RARαa binding in zebrafish (*danRer10*) were mapped to the mouse genome (*mm10*) using UCSC LiftOver for orthologous genomic regions that have conserved sequences (the *Alx1* and *Alx4* loci in Figure 6A) and Interspecies Point Projection (IPP) [25] for genomic regions that have diverged significantly (the *Alx3* locus in Figure 6A). To utilize the precomputed pairwise alignments file between *mm10* and *danRer11* provided by the authors of IPP, the RARαa ChIP‐seq peaks were first mapped to *danRer11* using LiftOver, and then projected to *mm10* using the IPP algorithm; the resulting genomic regions were mapped to *mm10* using LiftOver for visualization in the genome browser. The PCHi‐C results in tab‐separated text files were converted to BEDPE format and visualized in the IGV genome browser.

## 3 Results

### 3.1 Blocking RA Signaling Caused Midline Facial Cleft and Premaxillary Hypoplasia

**FIGURE 1:**
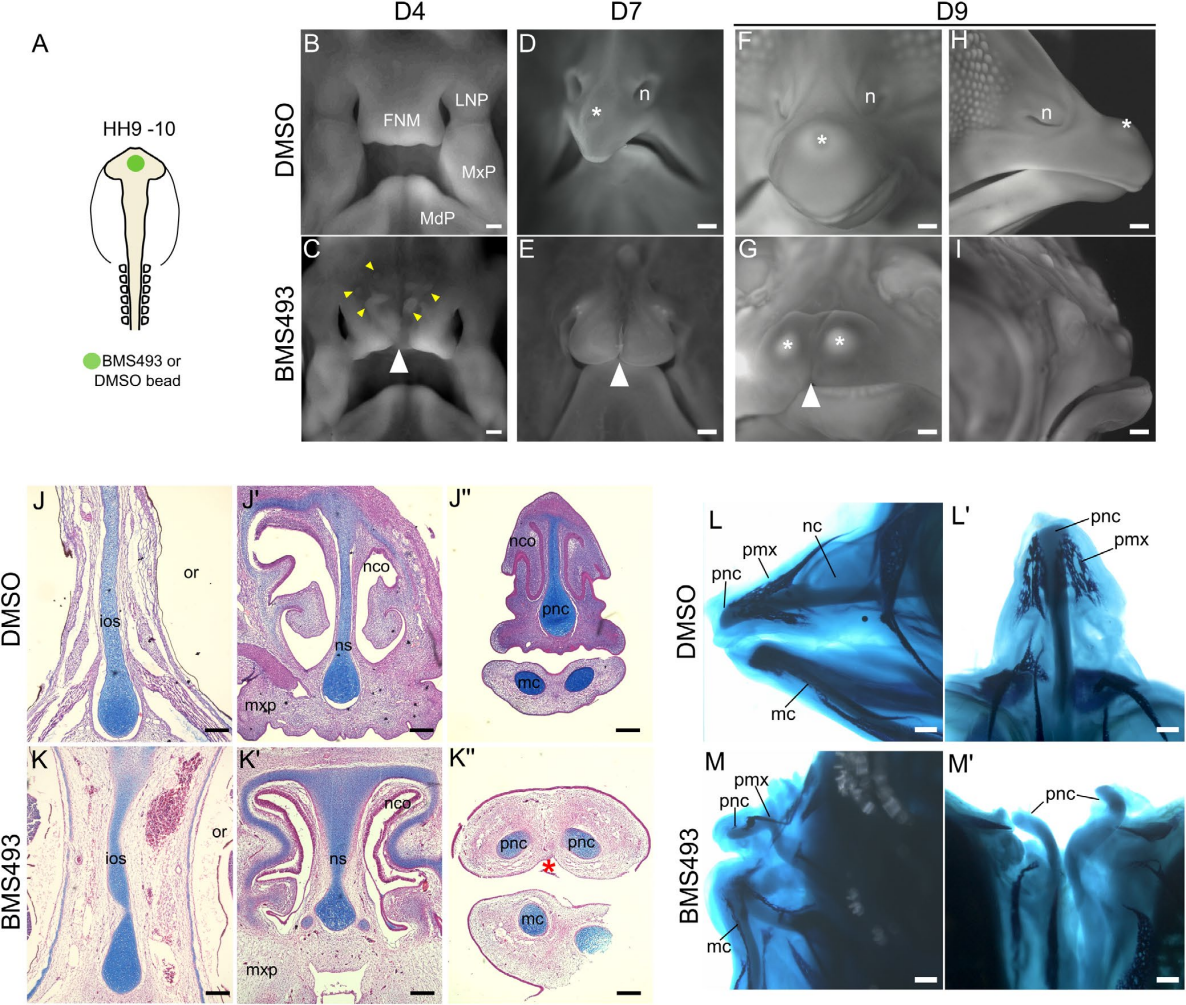
Blocking RA signaling caused midfacial clefting and premaxillary hypoplasia. (A) Schematic of bead implantation: beads (green) soaked with DMSO or BMS493 were implanted into the rostral forebrain of HH stage 9–10 chick embryos. (B, C) Four days after bead implantation, BMS493‐treated embryos (50/66, 75.8%) exhibited midfacial clefting (arrowhead) compared with DMSO‐treated controls (0/51), as visualized by whole‐mount DAPI staining. Yellow arrowheads in C indicate abnormal nodules in the FNM of BMS493‐treated embryos. (D, E) Whole‐mount DAPI staining of embryos 7 days after bead implantation. BMS493‐treated embryos (12/15, 80%) exhibited a midline cleft of the upper beak (arrowhead) and collapsed nostrils. (F–I) Whole‐mount frontal and lateral views of DMSO‐ and BMS493‐treated embryos 9 days after bead implantation. BMS493‐treated embryos (6/9, 66.7%) exhibited a truncated upper beak with a midline cleft (arrowhead) and duplicated egg teeth. (J–K″) Frontal sections of the heads from DMSO‐ and BMS493‐treated embryos shown in panels D and E. (L–M') Skeletal staining of the heads from DMSO‐ and BMS493‐treated embryos shown in panels F–I. L, M, lateral view; L', M', ventral view. White asterisk in D, F, G and H marks the egg tooth. Red asterisk in K″ marks the midline cleft of the upper beak. Scale bar, B–C, J–K“ 200 μm; D–I, L–M' 400 μm. FNM, frontonasal mass; LNP, lateral nasal process; MxP, maxillary process; MdP, mandibular process; n, nostril; ios, interorbital septum; or, orbit; nco, nasal conchae; ns, nasal septum; pnc, prenasal cartilage; mc, Meckel's cartilage; pmx, premaxilla; nc, nasal cartilage.

To understand the role of RA signaling during facial development, we investigated the impact of blocking RA signaling during early craniofacial development. Beads soaked in BMS493, an inverse pan‐RAR agonist, were implanted into the rostral forebrain of HH9‐10 chick embryos (Figure 1A). Four days after bead implantation, 50 of 66 (75.8%) BMS493‐treated chick embryos exhibited varying degrees of midfacial defects (Figure 1C), including hypoplasia, midline clefts, and abnormal nodules in the frontonasal mass (FNM)–the chick homologous structure of the mammalian MNP. These phenotypes were not observed in any of the DMSO‐treated control embryos (*n* = 51) (Figure 1B). Seven days after bead implantation, the upper beak was well developed in DMSO‐treated embryos (*n* = 11), whereas 12 of 15 (80%) BMS493‐treated chick embryos exhibited a midline cleft of the upper beak, collapsed nostrils, and loss of the egg tooth (Figure 1D,E). Histological analyses revealed that the BMS493‐treated embryos exhibited a discontinuous interorbital septum (Figure 1K), dorsal widening of the nasal septum (Figure 1K′), and bifurcated prenasal cartilage (Figure 1K”). Nine days post bead implantation, 6 of 9 (66.7%) BMS493‐treated embryos had a truncated upper beak with a midline cleft and duplicated egg teeth (Figure 1F–I). Consistent with the defects observed in the FNM, skeletal staining of embryos at this stage further showed that the development of premaxillary bone was severely compromised in BMS493‐treated embryos, accompanied by bifurcated prenasal cartilage (Figure 1L,M).

### 3.2 Blocking RA Signaling Caused Patterning Defects in the Frontonasal Neural Crest Cells

**FIGURE 2:**
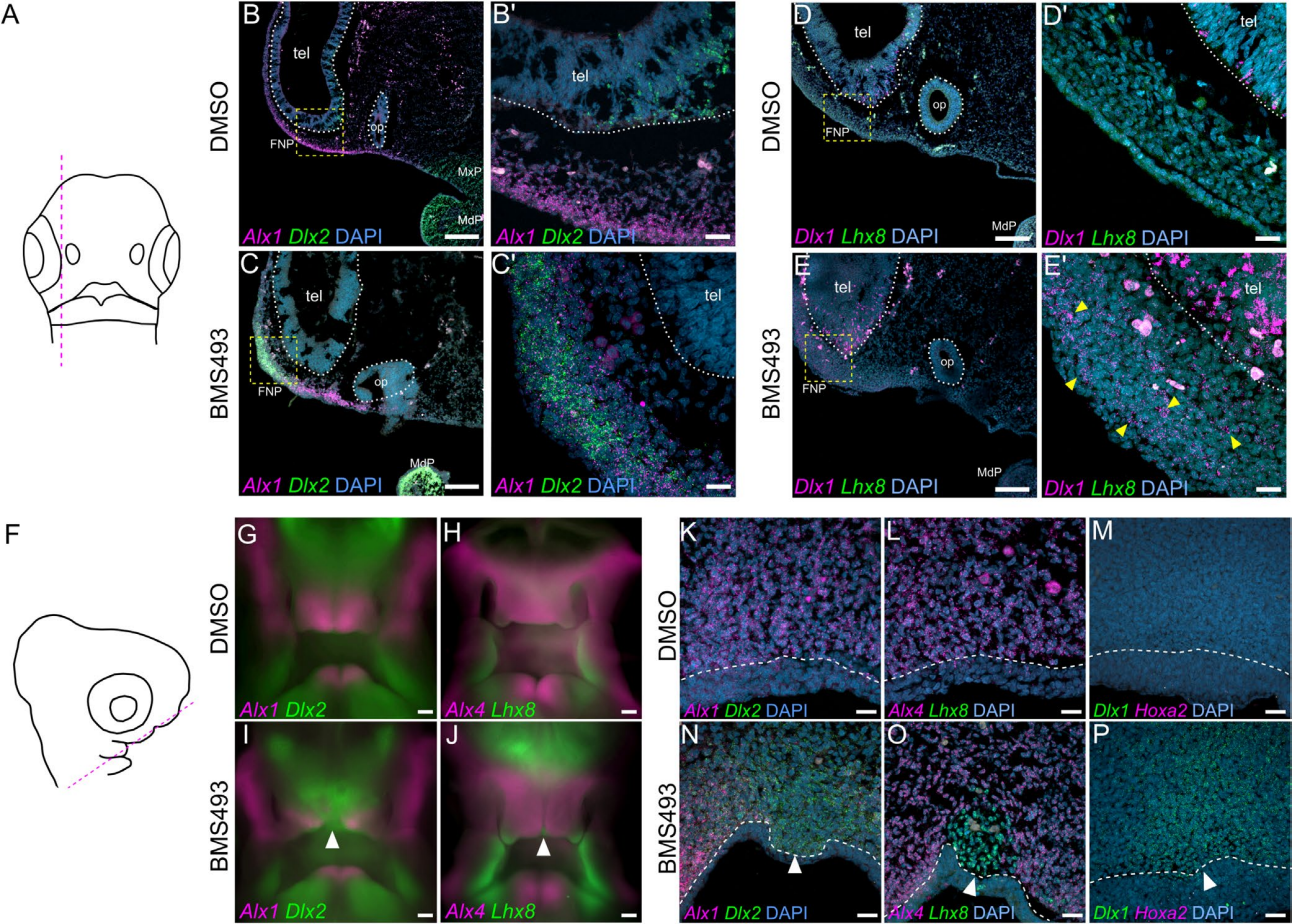
Blocking RA signaling caused patterning defects in the frontonasal neural crest cells. (A) Schematic illustration of the frontal view of the chick head 2 days after bead implantation. The dotted line indicates the cryosection plane for the images shown in B–E'. (B–E') Sagittal sections of *Alx1/Dlx2* and *Dlx1/Lhx8* HCR revealed ectopic *Dlx2* and *Dlx1* expression in the frontonasal NCCs of BMS493‐treated embryos (19/22, 86.4%) 2 days after treatment. B′–E' are magnified views of the yellow boxes in B‐E, respectively. Yellow arrowheads in E' indicate ectopic *Dlx1* expression in the frontonasal NCCs. (F) Schematic illustration of the sagittal view of the chick head 4 days after bead implantation. The dotted line indicates the cryosection plane for the images shown in K–P. (G–J) Whole‐mount HCR showed reduced frontonasal marker genes (*Alx1/4*) and ectopic pharyngeal arch marker genes (*Dlx2, Lhx8*) expression (white arrowheads) in the FNM of BMS493‐treated chick embryos (31/36, 86.1%) 4 days after treatment. (K–P) Coronal sections through the FNM showed decreased *Alx1/4* and ectopic expression of *Dlx1/2* and *Lhx8* in neural crest‐derived mesenchymal cells at the midline clefting site (white arrowheads) of BMS493‐treated chick embryos. The nuclei were counterstained with DAPI. Dotted lines delineate the boundary between the epithelium and mesenchyme. Scale bar, A‐D, E‐H 200 μm; A'–D′, I–N 20 μm. tel., telencephalon; FNP, frontonasal process; LNP, lateral nasal process; MxP, maxillary process; MdP, mandibular process; op, optic placode.

Since the defects induced by BMS493 are largely restricted to the frontonasal region, we ask whether there was a patterning defect induced by BMS493 during early facial development. We examined the expression of positional transcription factors during the patterning of the face in BMS493‐ and DMSO‐treated embryos by HCR, followed by cryosectioning along the indicated planes (Figure 2A,F).

Two days after treatment, DMSO‐treated embryos (*n* = 16) exhibited normal gene expression patterns, with *Alx1* expressed in the FNP while *Dlx1* and *Dlx2* were expressed in the PA1 NCCs (HH18‐19) (Figure 2B,B',D,D'). In contrast, although no morphological defects could be observed in BMS493‐treated embryos at this stage, ectopic expression of both *Dlx1* and *Dlx2* was detected in the frontonasal NCCs of these embryos (19 of 22, 86.4%) (Figure 2C,C',E,E'). This result suggests that a subpopulation of CNCCs that arrived at the FNP region adopted a pharyngeal arch identity instead of frontonasal identity.

Four days after treatment, the expression patterns of frontonasal marker genes (*Alx1/4*) and pharyngeal arch marker genes (*Dlx1/2*, *Lhx8*) remained normal in DMSO‐treated embryos (*n* = 25) (Figure 2G,H,K–M). Whereas in 31 of 36 (86.1%) BMS493‐treated embryos, we continued to observe ectopic expression of *Dlx1/2* and *Lhx8* in the CNCC‐derived mesenchymal cells of the FNM, concomitant with the loss of *Alx1* and *Alx4*, which are normally expressed in the FNM and the LNP (Figure 2I,J,N–P). The expression of *Hoxa2*, which is normally expressed in pharyngeal arches 2–6, was not observed in the FNM of any embryos (Figure 2M,P), suggesting that an ectopic PA1 identity was established in the CNCCs of the FNM in RA signaling‐deficient embryos. This erroneous PA1‐like identity in the FNM is likely responsible for the severe premaxillary defects of BMS493‐treated embryos and may also play a role in the midline clefting phenotype.

### 3.3 RA Regulates the Expression of *Alx1* and *Alx4* in Midfacial Development

A previous study showed that *Alx1* and *Alx4* are required for preventing ectopic PA1‐like identity in the frontonasal region [11]. Furthermore, mutations in Alx genes have been associated with midline facial clefting in humans and mouse [7, 26, 27]. We therefore analyzed whether Alx genes are the target genes and key downstream effectors of RA signaling during midfacial development.

**FIGURE 3:**
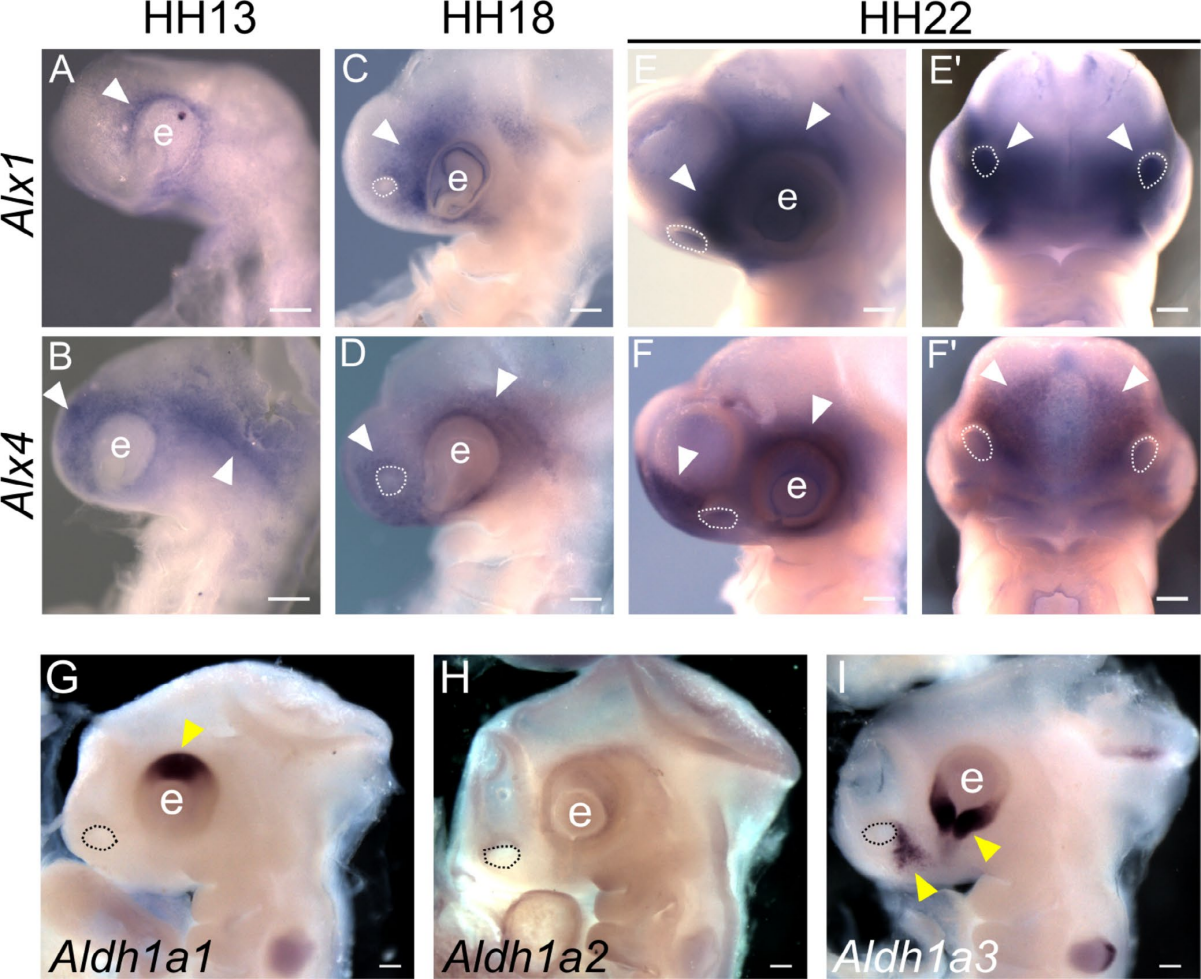
Expression patterns of Alx genes during frontonasal development. (A–H) Whole‐mount ISH showing lateral (A–F) and frontal (E', F′) views of *Alx1* and *Alx4* mRNA expression (blue/purple color) patterns in wildtype embryos at HH13 (A, B), HH18 (C, D), and HH22 (E–F′). White arrowheads indicate the expression domains of *Alx1* and *Alx4* in the FNP, periocular region and head mesenchyme of chick embryos. (G‐I) Whole‐mount ISH showing lateral views of *Aldh1a1/2/3* mRNA expression (blue/purple color) patterns in wildtype embryos at HH18. Yellow arrowheads indicate the expression domains of *Aldh1a1 and Aldh1a3* in the developing retina and nasal placode of chick embryos. Dotted circles outline the position of nasal pits. Scale bar, A–D, G–I, 100 μm; E–F′, 200 μm. e, eye.

The expression patterns of *Alx1* and *Alx4* were examined in chick embryos during facial development from HH13 to HH22 (Figure 3A–F') by whole‐mount ISH. At HH13, the expression of *Alx1* was first detected in the periocular region, whereas the expression of *Alx4* was found in both the periocular region and the head mesenchyme (Figure 3A,B). At HH18, while both Alx genes were expressed in the frontonasal and periocular region, *Alx1* expression was biased toward the emerging LNP, while *Alx4* showed a stronger expression in the emerging FNM (Figure 3C,D). At HH22, when all the major facial primordia had formed, both *Alx1* and *Alx4* were observed in the LNP, FNM, and the distal ends of the MxP and MdP (Figure 3E,F').

To identify the potential local source of RA, we examined the expression of Aldh1a genes, which encode enzymes responsible for the final step of RA synthesis [28] during the early patterning of the face at HH18. *Aldh1a1* expression was found in the dorsal side of the retina (Figure 3G), while *Aldh1a3* was detected in the ventral side of the developing retina as well as the nasal placode (Figure 3I). *Aldh1a2* expression appeared weak and non‐specific in the craniofacial region at this developmental stage (Figure 3H). Based on the expression patterns of Aldh1a genes, we proposed a model that local RA produced by the retina and nasal placode may induce the expression of Alx genes in the surrounding mesenchymal tissues.

**FIGURE 4:**
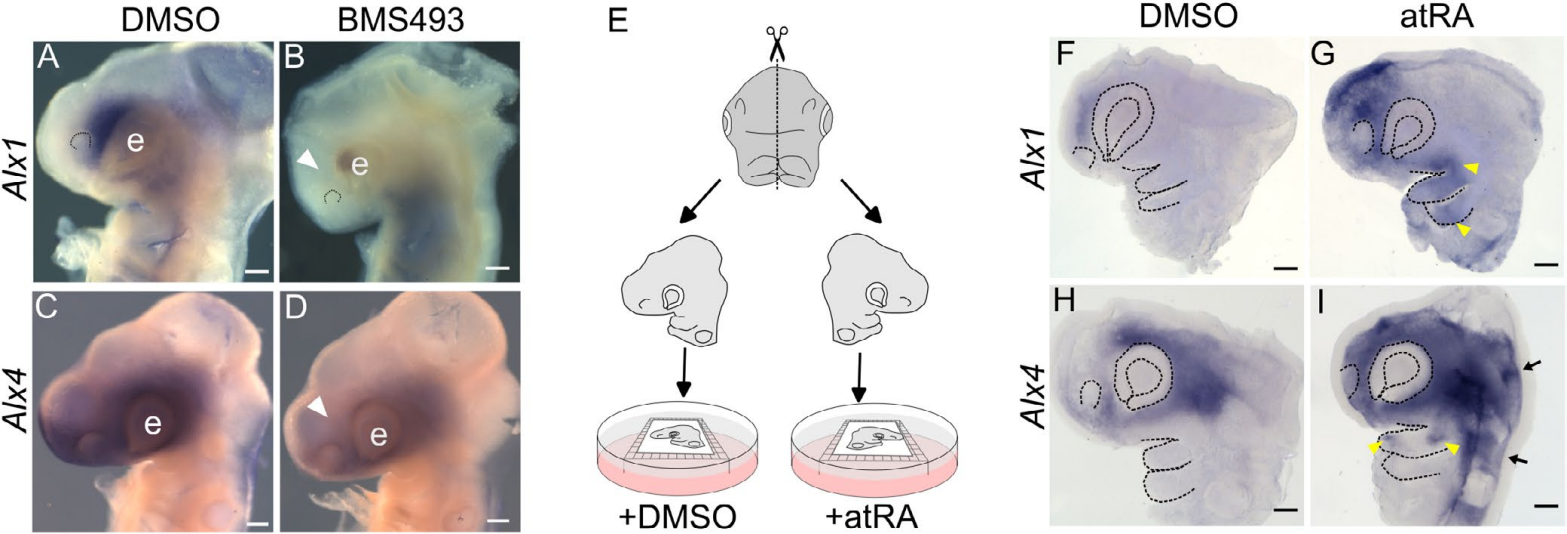
The craniofacial expression of *Alx1* and *Alx4* is regulated by RA signaling. (A–D) Both the expressions of *Alx1* and *Alx4* (purple staining) in the embryonic head were significantly reduced 2 days after implantation of BMS493‐soaked beads (26/28, 92.9%) compared to DMSO controls (0/22). Dotted circles outline the position of nasal pits. Arrowheads in (B, D) indicate the decreased expression of *Alx1* and *Alx4* in the FNP. e, eye. (E) Schematic diagram of the facial explant culture of chick embryos using the Trowell organ culture method. The heads of HH17‐18 embryos were bisected through the midline, placed on filter membranes and cultured at the liquid‐air interface. (F‐I) ISH of facial explants showed a dramatic increase in *Alx1* and *Alx4* expression in the frontonasal region of atRA‐treated explants (*n* = 13) compared to DMSO controls (*n* = 9) after 24 h of incubation, with a subset of atRA‐treated explants also exhibiting moderate ectopic expression in the PAs (*Alx1*: 5/9, *Alx4*: 1/4) (yellow arrowheads). Black arrows indicate increased *Alx4* expression in non‐NCC regions. The dotted lines outline the eye, nasal pit, and PAs. Scale bar, A–D, F–I, 200 μm.

To test whether Alx genes are regulated by RA, we examined the expression of *Alx1* and *Alx4* before the manifestation of BMS493‐induced phenotypes. Two days after bead implantation, we found that the expression of both *Alx1* and *Alx4* was dramatically reduced in BMS493‐treated embryos (26 of 28, 92.9%) compared to the DMSO‐treated controls (0 of 22) (Figure 4A–D). This finding echoes a previous report that the expression of *Alx1* and *Alx3* is reduced in *Rdh10*‐mutant embryos [16], although we could not investigate *Alx3* in chick embryos due to the loss of this gene in chicken [5].

To test the effects of excessive RA on Alx expression, we adopted the Trowell organ culture method (Figure 4E) to circumvent the secondary effects resulting from the inhibitory role of RA on CNCC delamination and migration [29]. After exposure to 1 μM of all‐*trans* RA (atRA) (or the same volume of DMSO in the control samples) for 24 h, RA‐treated explants exhibited dramatically increased expression of *Alx1* (8 of 9, 88.9%) and *Alx4* (4 of 4, 100%) in the frontonasal region compared to their DMSO‐treated controls (Figure 4F–I). Interestingly, in addition to the frontonasal region, *Alx4* expression was also markedly increased in non‐NCC regions (Figure 4I). In contrast to the consistently elevated expression of *Alx1* and *Alx4* in the frontonasal region, only a moderate level of ectopic *Alx1* or *Alx4* expression was observed in the PAs of a subset of RA‐treated facial explants (*Alx1*: 5 of 9, 55.6%; *Alx4*: 1 of 4, 25%), suggesting the existence of inhibitors of *Alx1/4* in the PAs (Figure 4G,I).

### 3.4 Cell‐Autonomous Role of RAR Signaling in CNCCs Patterning Through Alx Genes

Having established that RA signaling plays a critical role in regulating the expression of Alx genes during the patterning of the face, we ask whether this regulatory effect requires cell‐autonomous RAR signaling in CNCCs or depends on indirect, cell‐nonautonomous mechanisms involving signals from neighboring tissues, such as *Fgf8* and *Shh* [16, 17].

**FIGURE 5:**
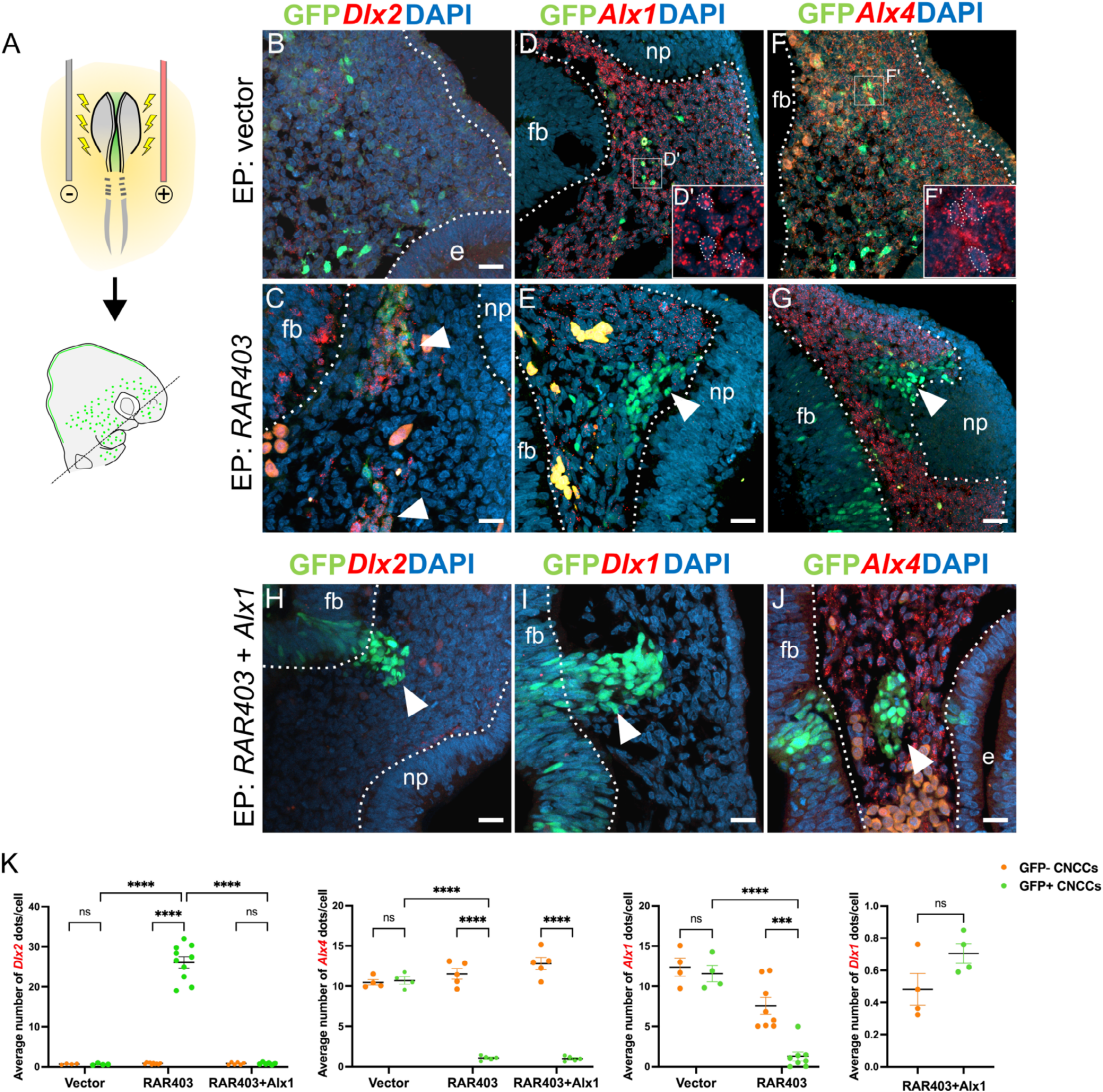
Cell‐autonomous requirement of RARs for the expression of Alx genes and patterning of CNCCs. (A) Schematic of RARα‐403‐myc‐GFP (*RAR403*) electroporation into one side of HH8‐HH8+ chick neural tubes. Embryos were harvested at 2 days post‐electroporation for cryosection. The dotted line indicates the section plane for images in B‐H. (B–G) HCR showing the effect of *RAR403* on the mRNA expression of *Alx1*, *Alx4* and *Dlx2*. Vector‐transfected CNCCs (nuclear GFP+) did not exhibit ectopic *Dlx2* mRNA and had similar expression levels of *Alx1* and *Alx4* compared to neighboring cells (B, D, F). B and F represent the same biological sample co‐labeled with *Dlx2* and *Alx4* HCR probes. Inserts D′ and F′ are magnified views of the boxes in D and F, respectively. Dotted lines in D′ and F′ delineate GFP+ transfected cells. *RAR403*‐transfected CNCCs (C, E, G) in the FNP exhibited increased aggregation, ectopic expression of *Dlx2* (*n* = 10) and lost expression of both *Alx1* (*n* = 8) and *Alx4* (*n* = 5) compared to the neighboring non‐transfected cells and vector‐transfected CNCCs (B, D, F). Dotted lines indicate the boundaries between mesenchyme and epithelium or neural epithelium. Arrowheads indicate the aggregates of *RAR403*‐transfected CNCCs. (H‐J) Co‐transfection with a chick *Alx1* expression construct suppressed the *RAR403*‐induced ectopic expression of *Dlx2* (*n* = 6) and *Dlx1* (*n* = 4), but did not restore the lost *Alx4* expression (*n* = 5) in transfected CNCCs. (K) Quantification of HCR‐positive dots for *Dlx2*, *Alx4*, *Alx1*, and *Dlx1* in vector‐transfected, *RAR403*‐transfected, *RAR403* and *Alx1* co‐transfected, and non‐transfected CNCCs. Three sections from each embryo were analyzed. ***p < 0.001; ****p < 0.0001. Statistical analyses were performed using two‐way ANOVA and an unpaired *t*‐test. Scale bar, B–J, 20 μm. e, eye; fb, forebrain; np, nasal pit.

We reasoned that if the regulation of *Alx1/4* mainly relies on cell‐autonomous RAR signaling, blocking RARs in a subset of frontonasal NCCs will cause the loss of *Alx1/4* and the ectopic expression of PA1 markers specifically within this subset of cells. To test this, we electroporated a dominant‐negative form of *hRARα* (*RAR403*) [22] into HH8‐HH8+ chick neural tubes (Figure 5A).

The electroporation resulted in a mosaic population of transfected CNCCs, labeled by the GFP signal, that migrated into the facial primordia. HCR results showed that within the FNP, *RAR403*‐transfected CNCCs, but not their adjacent non‐transfected cells, ectopically expressed the PA1 marker gene *Dlx2* (*n* = 10) (Figure 5B,C). In addition, *RAR403*‐transfected CNCCs in the FNP lost expression of *Alx1* (*n* = 8) and *Alx4* (*n* = 5), whereas their surrounding non‐transfected cells remained unaffected (Figure 5D–G). These changes were quantified by counting the number of HCR dots (Figure 5K). Based on these results, we concluded that cell‐autonomous RAR signaling regulates Alx gene expression and positional identity in CNCCs.

In contrast to the robust ectopic expression of *Dlx2* when *RAR403* alone was transfected, co‐transfection with the chick *Alx1* expression construct suppressed the *RAR403*‐induced ectopic expression of both *Dlx2* (*n* = 6) and *Dlx1* (*n* = 4) (Figure 5H,I,K). However, *Alx1* co‐transfection failed to restore *Alx4* expression in *RAR403*‐transfected CNCCs (*n* = 5) (Figure 5J,K). These results suggest that Alx genes function as important downstream effectors of RA signaling during CNCCs patterning, whereas *Alx1* alone is insufficient to fully compensate for the molecular consequences of RA inhibition.

### 3.5 *Alx1 and Alx4* Are Likely Direct Downstream Targets of RA Signaling in Frontonasal NCCs

**FIGURE 6:**
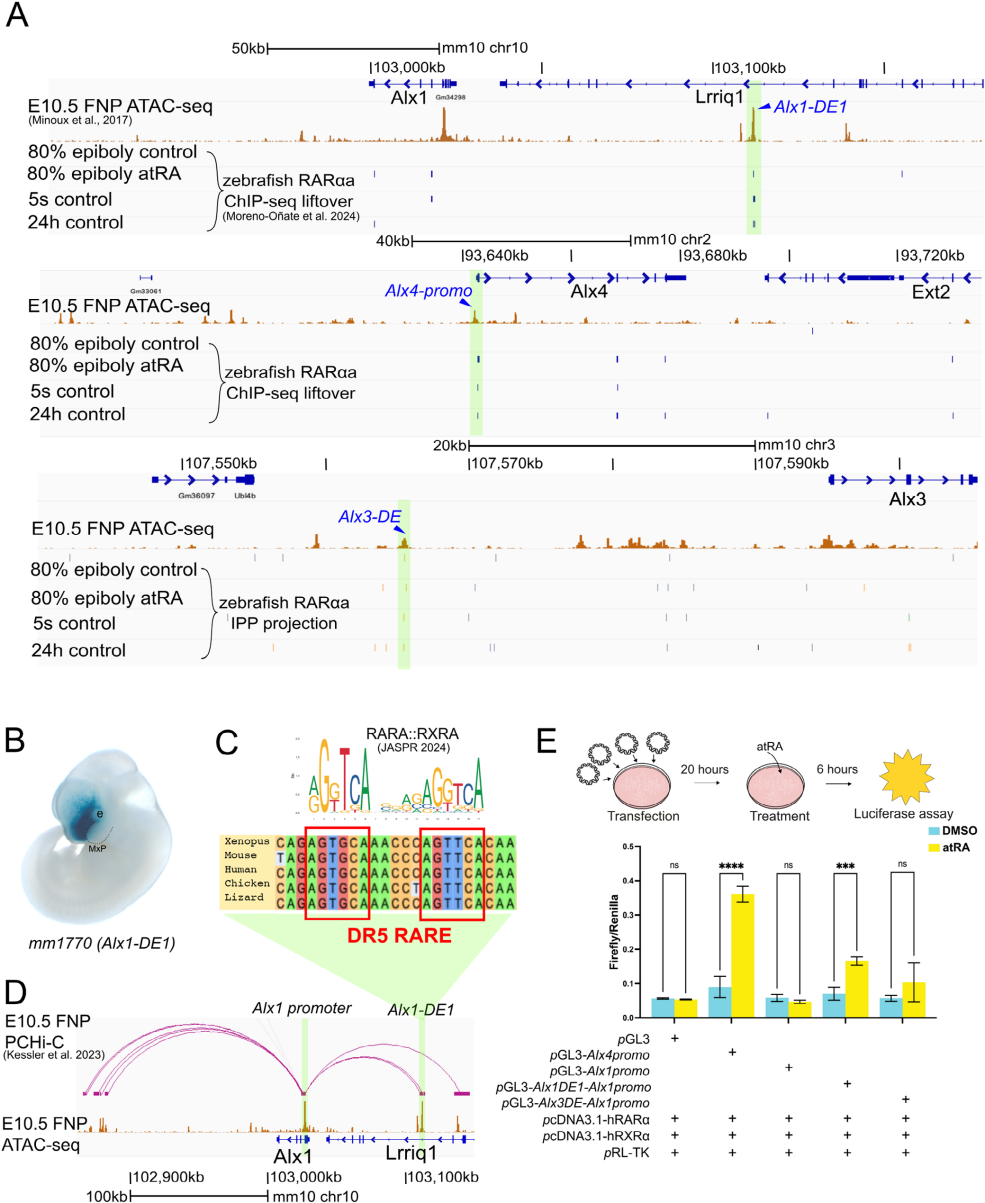
*Alx1* and *Alx4* are likely direct targets of RA signaling. (A) RARαa ChIP‐seq binding peaks from zebrafish embryos were mapped to the mouse *mm10* genome using UCSC LiftOver or interspecies point projection (IPP), and aligned with ATAC‐seq signal tracks from E10.5 mouse FNP. The genomic regions at the *Alx1* and *Alx4* loci were mapped to the mouse genome by UCSC LiftOver; the genomic regions at the *Alx3* locus were mapped to the genome by IPP. Regions marked in orange are determined as “indirectly conserved” by IPP. Based on the mapping results, one putative *Alx1* distal enhancer (*Alx1‐DE1*), one putative *Alx3* distal enhancer (*Alx3‐DE*), and the promoter of *Alx4* (*Alx4‐promo*) were identified as potential RA‐responsive CREs. (B) Transgenic reporter activity (blue staining) of *mm1770* recapitulates the expression pattern of *Alx1*. This image is from the VISTA genome browser and used with permission. (C) The evolutionarily conserved DR5 RARE in *Alx1‐DE1*. Red boxes highlight the DR5 RARE sequences. (D) *Alx1‐DE1* physically interacts with the promoter of *Alx1*. Purple arches indicate long‐range interactions between the *Alx1* promoter and chromatin regions including *Alx1‐DE1*. (E) *Alx1‐DE1* and *Alx4‐promo* are RA‐responsive. The schematic diagram illustrates the design of the luciferase assay. Results were presented as the ratio of firefly to *Renilla* luciferase activity (means ± SEM). Statistical significance was determined using two‐way ANOVA. ***p < 0.001, ****p < 0.0001. *Alx1promo*, the promoter of *Alx1*; *Alx4promo*, the promoter of *Alx4*.

RA signaling regulates its target genes through the interactions between liganded RAR/RXR heterodimers with RA‐responsive elements (RARE) in the regulatory sequences in the genome. To identify the *cis‐*regulatory elements (CREs) of Alx genes that are responsive to RA, we reanalyzed public RARαa ChIP‐seq data from zebrafish embryos [30]. We mapped RARαa binding peaks onto the mouse genome (*mm10*) using UCSC liftover or interspecies point projection (IPP), a synteny‐based algorithm [25]. By integrating the mapping result with ATAC‐seq data from E10.5 mouse FNP [31], we identified one putative *Alx1* distal enhancer (*Alx1‐DE1*), one putative *Alx3* distal enhancer (*Alx3‐DE*), and the promoter of *Alx4* as candidate RA‐responsive CREs (Figure 6A). Among these, *Alx1‐DE1* was recently reported as an important enhancer for the expression of *Alx1* in the FNP. Deletion of this enhancer specifically reduces *Alx1* expression in the CNCCs‐derived frontonasal mesenchyme and leads to frontonasal defects that partially phenocopy those observed in *Alx1*‐deficient mice [32].

We employed luciferase assays to test whether the mouse versions of these CREs are indeed RA‐responsive. To ensure receptor availability, we co‐transfected HEK293T cells with constructs of *hRARα* and *hRXRα* when performing the luciferase assay. Six hours after treating the transfected cells with 0.5 μM of atRA, both *Alx1‐DE1* and the *Alx4* promoter exhibited a robust and consistent increase in their activity, while *Alx3‐DE* did not respond to the RA treatment (Figure 6E).

Further analysis revealed an evolutionarily conserved retinoic acid responsive element (RARE) consisting of a direct repeat of the RARE half sites, RGKKSA [33], separated by 5 base pairs (DR5 RARE), in *Alx1‐DE1* (Figure 6C). Querying the VISTA enhancer browser [34, 35], we found that *Alx1‐DE1* overlapped with enhancer *mm1770*, which exhibits enhancer activity similar to the expression pattern of *Alx1* (Figure 6B). Whereas *Alx1‐DE1* is located within the intronic region of the gene *Lrriq1*, our reanalysis of the public PCHi‐C data [36] indicates that it interacts with the *Alx1* promoter in the developing FNP (Figure 6D).

A close inspection of the *Alx4* promoter sequence revealed two possible non‐canonical RAREs: one is the inverse repeat of the RARE half sites, 9 base pairs apart (IR9), and the other is DR6. Among these motifs, IR9 is shown to be overrepresented in RAR binding peaks [37]. We did not identify any canonical RAREs in the region we tested.

These results collectively indicate that *Alx1* and *Alx4* are likely direct target genes of RA signaling in CNCCs. Although we did not detect RA responsiveness of *Alx3‐DE* by our luciferase assay, we cannot exclude the possibility of *Alx3* also being a direct target of RA signaling in mammals. Genomic profiling of RAR binding sites in mammalian CNCCs can help us answer this question.

## 4 Discussion

RA is involved in a multitude of biological processes, including various stages of craniofacial development, as reviewed in [38]. Studies in animals have shown that precisely controlled RA signaling is crucial for proper craniofacial morphogenesis. At E9, when widespread RA reporter signals are detected across the FNP region [16, 39], the teratogenic effect of excessive RA specifically spares FNP‐derived tissues while causing profound defects on PA1 derivatives [40]. Conversely, inhibition of RA signaling disproportionally disrupts the development of FNP‐derivatives in mouse [16] and chicken [17], leading to severe hypoplasia and clefting of the midface. These observations suggest that RA signaling is particularly important for the normal development of the FNP. Our findings offer novel mechanistic insights into the roles of RA signaling during this developmental process.

The midfacial phenotypes of our BMS493‐treated chick embryos largely recapitulated those reported in *CreERT2: Rdh10* ^*flox/flox*^ mouse embryos [16], but were significantly milder than the phenotypes reported by Schneider et al. [17], which included cyclopia and agenesis of the upper jaw. This difference in severity was probably due to the use of different RA inhibitors; the combinatory use of RAR and RXR inhibitors by Schneider et al. [17] might have also led to inhibition of RA‐independent functions of RXR genes, thereby leading to a more dramatic phenotype.

Consistent with the reduction of *Alx1* and *Alx3* expression observed in *CreERT2: Rdh10* ^*flox/flox*^ embryos [16], we observed significant down‐regulation of both *Alx1* and *Alx4* in BMS493‐treated chick embryos. We further showed that this loss of Alx expression resulted in patterning defects in the frontonasal region. Given that the midfacial clefting in the *mouse model* was attributed to excessive Shh signaling [16], future studies should determine whether these patterning defects are also a contributing factor to RA‐associated midfacial clefting independent of Shh signaling.

We observed that *RAR403*‐transfected cells tend to form aggregates at the frontonasal region, indicating changes in cell migration behavior due to RA inhibition. Therefore, one plausible alternative explanation for the patterning defects is that RA inhibition may have caused a subset of CNCCs destined for PA1 to be “misrouted” into the frontonasal region.

However, several lines of evidence argue against this interpretation. First, this theory does not readily account for the global reduction of the expression of Alx genes after BMS493 treatment, the genes that play an important role in coding the frontonasal identity of CNCCs [11, 41]. Second, the positional identity of post‐migratory CNCCs of the face is believed to be plastic, influenced by local environmental cues [31, 42]; small groups of PA1 CNCCs straying into the frontonasal region would likely have adopted a new identity under the influence of the local environment. Furthermore, the ectopic PA1 marker‐expressing cells were mostly localized to the facial midline; they were not found in regions close to PA1. Based on these reasons, a cell identity change is more likely to be the main contributing factor underlying the facial patterning defects observed in this study.

Regarding the midline facial clefting phenotype, altered cell migration, adhesion, or survival—which may occur secondary to or independent of changes in cell identity—could also be important contributing factors.

Mechanistically, we showed that RA signaling directly regulates *Alx1* expression through the evolutionarily conserved RA‐responsive enhancer *Alx1‐DE1*. While *Alx1‐DE1* is located within the intronic region of the gene *Lrriq1*, it forms long‐range interactions with the promoter of *Alx1* in E10.5 mouse FNP, indicating the target gene of this enhancer is *Alx1*, rather than *Lrriq1*, at least in early craniofacial development. A recent report characterized *Alx1‐DE1* and revealed its role as a critical tissue‐specific enhancer of *Alx1* regulated by *Twist1* during craniofacial morphogenesis [32]. *Alx1‐DE1* is therefore likely an important regulatory node where multiple inputs are integrated to control the expression of *Alx1*.

Another recent study suggested that in the context of periocular mesenchyme, RA signaling directly regulates the expression of *Alx1* through a putative RARE [43]. However, this study did not test the RA‐responsiveness of the *Alx1* promoter, and our own study failed to observe consistent RA‐responsiveness for the mouse *Alx1* promoter. With regard to the mouse *Alx4* promoter, despite the robust RA‐responsiveness we observed by luciferase assay, we did not identify any canonical RAREs. Although non‐canonical motifs DR6 and IR9 were identified, these sequences are not conserved between human and mouse. One possibility is that, although different species might have distinct configurations of RAREs in their *Alx4* promoters, these RAREs confer similar RA‐responsiveness.

While our findings and bioinformatics analyses strongly indicate that *Alx1/4* are direct target genes of RA signaling, direct evidence, such as high quality ChIP‐seq or CUT&RUN on frontonasal NCCs, is essential to conclusively establish this regulatory link. Futhermore, whether *Alx3* is a direct target of RA signaling should also be investigated in future studies using mouse models or human pluripotent stem cell (hPSC) – derived NCCs.

Although RA signaling is indispensable for the expression of *Alx1/4* in frontonasal NCCs, RA alone is likely insufficient for maintaining robust *Alx1/4* expression. Despite the significantly increased *Alx1/4* expression in the FNP region of RA‐treated facial explants, only a subset of samples showed clear ectopic *Alx1/4* expression in the PAs under the same culture conditions. In addition, the requirement for combined Noggin and RA treatment to successfully reprogram chick MxP into FNP [4] suggests that BMP signaling, which is antagonized by Noggin, may restrict the expression of *Alx* genes in PA1. Furthermore, other neural crest transcription factors also play a role in regulating the expression of *Alx* genes, such as *Tfap2* paralogs and *Twist1* [32, 44, 45]. Further investigation is warranted to understand how these signaling inputs are integrated within CNCCs to precisely regulate Alx gene expression and confer FNP identity.

Disruption of RA signaling by ethanol has been hypothesized to be a pathogenic mechanism of fetal alcohol syndrome (FASD). This hypothesis was well supported by animal studies (Reviewed in [46]). Our findings imply that Alx genes might be involved in the pathogenesis of the craniofacial phenotypes in FASD patients, especially in cases with midfacial clefting [47]. This resonates with a recent report that losing *alx1* made zebrafish embryos more vulnerable to craniofacial and ocular defects caused by alcohol exposure [10]. Whether mutations in human ALX genes predispose individuals to alcohol‐related craniofacial defects remains an important question for future investigation.

## Author Contributions

Shuxuan Wu contributed to conception, design, data acquisition, analysis and interpretation, drafted and critically revised the manuscript. Yifan Lu contributed to data acquisition and critically revised the manuscript. Yixin Tu contributed to data acquisition and critically revised the manuscript. Yuke Xi contributed to data acquisition and critically revised the manuscript. Qianxue Wan contributed to data acquisition. Minghui Yue contributed to data analysis and interpretation, and critically revised the manuscript. Huan Liu contributed to data analysis and interpretation, and critically revised the manuscript. Zhaoming Wu contributed to conception, design, data acquisition, analysis and interpretation, drafted and critically revised the manuscript. All authors gave final approval and agreed to be accountable for all aspects of the work.

## Funding

The authors disclosed receipt of the following financial support for the research, authorship, and/or publication of this article: This work was supported by the National Natural Science Foundation of China (NSFC) grant number 82301011 and Xuzhou Basic Research Program grant number KC23067.

## Conflicts of Interest

The authors declare no conflicts of interest.

## Supporting information


**Table S1:** List of oligonucleotide probes used for in situ Hybridization Chain Reaction.

## Data Availability

The data that support the findings of this study are available in the Materials and Methods, Results and Supporting Information of this article.
