# Supplementary material for: Retinoic Acid Informs the Positional Identity of Frontonasal Neural Crest Cells Through Alx Family of Transcription Factors
Source: FASEB J. 2026 Jun 9;40(12):e72039. doi: 10.1096/fj.202601339R (PMC13249048; doi:10.1096/fj.202601339R)
Supplement: Supplementary file 1 — Table S1: List of oligonucleotide probes used for in situ Hybridization Chain Reaction. [file FSB2-40-e72039-s001.docx]

**Supplementary Table 1. List of primers used for *in situ* Hybridization chain reaction.**

**Chick *Alx4* HCR probe**

| Name | Sequences |
| --- | --- |
| cAlx4-B2-1P1 | CCTCGTAAATCCTCATCAaaAAACACTGCACACCTAAACCGATAT |
| cAlx4-B2-1P2 | TTCTTTCTTTCTCCCAGTCTCTCTTaaATCATCCAGTAAACCGCC |
| cAlx4-B2-2P1 | CCTCGTAAATCCTCATCAaaCCTAGCTCTACCTACCTACCTAGCG |
| cAlx4-B2-2P2 | CCTTCAAAAAATCAGTAGTACAAAAaaATCATCCAGTAAACCGCC |
| cAlx4-B2-4P1 | CCTCGTAAATCCTCATCAaaTTTCTGTTGTGTCAGTCAGTGCAGC |
| cAlx4-B2-4P2 | GTTTAATTTCTAATTCGGTCTGTTTaaATCATCCAGTAAACCGCC |
| cAlx4-B2-5P1 | CCTCGTAAATCCTCATCAaaTTTGTTTGGAGAACGTTTGACAAGG |
| cAlx4-B2-5P2 | TCCAGGAAACTACCTTTTTGGCTTTaaATCATCCAGTAAACCGCC |
| cAlx4-B2-7P1 | CCTCGTAAATCCTCATCAaaAGATGCTCGGAGGAGTTAAAGGGCT |
| cAlx4-B2-7P2 | CAGCTGTGCTCCAGCACGCACAGCAaaATCATCCAGTAAACCGCC |
| cAlx4-B2-8P1 | CCTCGTAAATCCTCATCAaaTTTGCAGATGCTGCCAATGCATGCA |
| cAlx4-B2-8P2 | GGGGAGCCGTGGGATGTTCACCGCTaaATCATCCAGTAAACCGCC |
| cAlx4-B2-16P1 | CCTCGTAAATCCTCATCAaaGGAGCAGAGCTCTGCACTTGCAGTG |
| cAlx4-B2-16P2 | TGGTGAATGGCAGCAGAGTGGGGCAaaATCATCCAGTAAACCGCC |
| cAlx4-B2-18P1 | CCTCGTAAATCCTCATCAaaTTCATCTCGTGAGCCGTTATGCAGC |
| cAlx4-B2-18P2 | ATGCATGAGGAGGAGAGGATGCAGCaaATCATCCAGTAAACCGCC |
| cAlx4-B2-21P1 | CCTCGTAAATCCTCATCAaaTGGAAAAGCGCCTTTCCCTCTGCAT |
| cAlx4-B2-21P2 | TGAGTTCTGGGAGGCTGAGCGCCGTaaATCATCCAGTAAACCGCC |
| cAlx4-B2-24P1 | CCTCGTAAATCCTCATCAaaATTTTTAGCACCTGAAGCACATTTT |
| cAlx4-B2-24P2 | ATCCAGAGCTATTCGGTAATCGGCAaaATCATCCAGTAAACCGCC |
| cAlx4-B2-25P1 | CCTCGTAAATCCTCATCAaaTGGAACGTGAGTCTGGTTTGCTTCT |
| cAlx4-B2-25P2 | TCTGATAACTCAGCTGAACGTTCAGaaATCATCCAGTAAACCGCC |
| cAlx4-B2-28P1 | CCTCGTAAATCCTCATCAaaCATCGTGACATTTGGTTGTAGCCTG |
| cAlx4-B2-28P2 | ATTTTTATATGGTATGCAGTTCACCaaATCATCCAGTAAACCGCC |
| cAlx4-B2-32P1 | CCTCGTAAATCCTCATCAaaATTGAATGGCTTGAGAGAGGTTTGG |
| cAlx4-B2-32P2 | TGCAATTATCCTTGATGCCATGACAaaATCATCCAGTAAACCGCC |
| cAlx4-B2-33P1 | CCTCGTAAATCCTCATCAaaAGAGAAAACAAGTTTTGGTGTTTCC |
| cAlx4-B2-33P2 | GGGGAAAACCATGGGTGGGACTTCAaaATCATCCAGTAAACCGCC |
| cAlx4-B2-39P1 | CCTCGTAAATCCTCATCAaaCAGCCATCATTTCATATGCATATCT |
| cAlx4-B2-39P2 | CTTCTGCAAGGTGGCAGTAGGGCCAaaATCATCCAGTAAACCGCC |

**Chick *Alx1* HCR probe**

| Name | Seq |
| --- | --- |
| cAlx1-B2-1P1 | CCTCGTAAATCCTCATCAaaCTGGAAGACTTTCTCCAGTTCCTCC |
| cAlx1-B2-1P2 | CACGTAGACATCAGGGTAATGAGTTaaATCATCCAGTAAACCGCC |
| cAlx1-B2-2P1 | CCTCGTAAATCCTCATCAaaTCTCCTCTTCTTACTGCTGGAGACA |
| cAlx1-B2-2P2 | CTGCAAACTGGTGAAAGTTGTTCTGaaATCATCCAGTAAACCGCC |
| cAlx1-B2-3P1 | CCTCGTAAATCCTCATCAaaCAGTTCCCCCTTCTCCTGCATCCCC |
| cAlx1-B2-3P2 | GCTGTCACACTTATCACCCAGTTCAaaATCATCCAGTAAACCGCC |
| cAlx1-B2-4P1 | CCTCGTAAATCCTCATCAaaTGTCCAAGGGCCTGTTGAGCTCTGT |
| cAlx1-B2-4P2 | CCGGAGACATCCTCAGATTGTTGCAaaATCATCCAGTAAACCGCC |
| cAlx1-B2-5P1 | CCTCGTAAATCCTCATCAaaCCATAGTTCACGTTGTTGTCTTGGC |
| cAlx1-B2-5P2 | AGAGGCTGTCCTTCCACTTTAGTAAaaATCATCCAGTAAACCGCC |
| cAlx1-B2-6P1 | CCTCGTAAATCCTCATCAaaCTCAGCCCTTTGCAGAGGGTTGAAG |
| cAlx1-B2-6P2 | TGATGTCCTGTCCAGCCTCACATGAaaATCATCCAGTAAACCGCC |
| cAlx1-B2-7P1 | CCTCGTAAATCCTCATCAaaGCTATAAAAGGACTCATTGTCCAAA |
| cAlx1-B2-7P2 | CTGCACACATTTGCTGCCTGACGTTaaATCATCCAGTAAACCGCC |
| cAlx1-B2-8P1 | CCTCGTAAATCCTCATCAaaTGCTCCCATGTAAAAGTCACTGTTC |
| cAlx1-B2-8P2 | TTCCATAACGTGCTCCAAAGTGCCTaaATCATCCAGTAAACCGCC |
| cAlx1-B2-9P1 | CCTCGTAAATCCTCATCAaaATGCTCTTTGGCTTTCATCCGTAGA |
| cAlx1-B2-9P2 | TTACATAGCCCAGGAAATATTGGCAaaATCATCCAGTAAACCGCC |
| cAlx1-B2-10P1 | CCTCGTAAATCCTCATCAaaCCGGCTTGGTTTCAAAAGCATGTCC |
| cAlx1-B2-10P2 | CAATGCTGGAAGATCTCCTTTCAAAaaATCATCCAGTAAACCGCC |
| cAlx1-B2-11P1 | CCTCGTAAATCCTCATCAaaGAAAAAATTATTGAGAGGCACATGG |
| cAlx1-B2-11P2 | GGTTGCCCCAGAAAGTAAAGAGTCAaaATCATCCAGTAAACCGCC |
| cAlx1-B2-12P1 | CCTCGTAAATCCTCATCAaaTGTGTAGCCAGAATCTGTCCGGGGT |
| cAlx1-B2-12P2 | GAACTGATTCTGGTGGTTTGAAAAGaaATCATCCAGTAAACCGCC |
| cAlx1-B2-13P1 | CCTCGTAAATCCTCATCAaaATCTCGTGGTATCATGCAGGAAGTA |
| cAlx1-B2-13P2 | GGAATAAGGTGTCATACAGGAGGAAaaATCATCCAGTAAACCGCC |
| cAlx1-B2-14P1 | CCTCGTAAATCCTCATCAaaCCACAGATTGTTCTGAATCTGAGGA |
| cAlx1-B2-14P2 | CACAGAACCACTAGCGGTGTTCCCCaaATCATCCAGTAAACCGCC |

**Chick *Dlx2* HCR probe**

| Name | Seq |
| --- | --- |
| cDlx2-B3-1P1 | GTCCCTGCCTCTATATCTttGCGTAGCCCGGGTCGTAGCCCGGCT |
| cDlx2-B3-1P2 | TAGGGCCCGTAGGAGCCGTACGCGGttCCACTCAACTTTAACCCG |
| cDlx2-B3-2P1 | GTCCCTGCCTCTATATCTttGGCTGCCGGTTGGCGTAGTAGCTGC |
| cDlx2-B3-2P2 | TAGGGGCAGGGCCCCGGCGGGCCGTttCCACTCAACTTTAACCCG |
| cDlx2-B3-3P1 | GTCCCTGCCTCTATATCTttTGCTGCTGATAGCCGCCGGCCGAGA |
| cDlx2-B3-3P2 | GGGGACTCCTGCGGCTTGTGCAGGCttCCACTCAACTTTAACCCG |
| cDlx2-B3-4P1 | GTCCCTGCCTCTATATCTttCTCGGCAGGACGCCGGTCATCCCGA |
| cDlx2-B3-4P2 | TGGCTGGCGTGCATGTCCGGCGCCGttCCACTCAACTTTAACCCG |
| cDlx2-B3-5P1 | GTCCCTGCCTCTATATCTttTAATCTCTGCTCGCTGTAAAAGAAA |
| cDlx2-B3-5P2 | CACCGTGGAAACAATAATTTATTTAttCCACTCAACTTTAACCCG |
| cDlx2-B3-6P1 | GTCCCTGCCTCTATATCTttATAACCAAAATACCCTTTGTTTGTT |
| cDlx2-B3-6P2 | AAAGAGCGCTGAAGTTTTCCGTAATttCCACTCAACTTTAACCCG |
| cDlx2-B3-7P1 | GTCCCTGCCTCTATATCTttCGCGCTGGCAGCCACGTGCCGAGGG |
| cDlx2-B3-7P2 | TTTGATTCGCCTCACGGCTCTCAGCttCCACTCAACTTTAACCCG |
| cDlx2-B3-8P1 | GTCCCTGCCTCTATATCTttAAATTATTTATCTTTTTTTGTTTGT |
| cDlx2-B3-8P2 | ACGCTCCGGCTTTAAACCTACATATttCCACTCAACTTTAACCCG |
| cDlx2-B3-9P1 | GTCCCTGCCTCTATATCTttGTCCCCAACATCAGAATGAGGTCTT |
| cDlx2-B3-9P2 | TATTTGTTTTTGTTCTCTTTTTGTTttCCACTCAACTTTAACCCG |
| cDlx2-B3-10P1 | GTCCCTGCCTCTATATCTttCGCCGCCGCCGCCGCCGCCGCCACC |
| cDlx2-B3-10P2 | AATGATCCGCGGTCCCCTAGAAGATttCCACTCAACTTTAACCCG |

**Chick *Dlx1* HCR probe**

| Name | Sequence |
| --- | --- |
| cDlx1-B2-1P1 | CCTCGTAAATCCTCATCAaaCCTGTTCAGAGCCTGCAGCTGCAAA |
| cDlx1-B2-1P2 | GGCCAGGTACTGGGTTTGCTGGAACaaATCATCCAGTAAACCGCC |
| cDlx1-B2-2P1 | CCTCGTAAATCCTCATCAaaTTTTTCCCTTTCCCATTAAAGCGAA |
| cDlx1-B2-2P2 | GAATAAATAGTCCTGGGTTTGCGGAaaATCATCCAGTAAACCGCC |
| cDlx1-B2-3P1 | CCTCGTAAATCCTCATCAaaTCCGATTCGGCCCCTGTCTCCTCCA |
| cDlx1-B2-3P2 | TCCCCTCCTTCTACCACAGTGCTTTaaATCATCCAGTAAACCGCC |
| cDlx1-B2-4P1 | CCTCGTAAATCCTCATCAaaGGGTAGGGCTGCACTGAACTGATGT |
| cDlx1-B2-4P2 | CGGGGCTGGGCCAGGCCGGAGCTGTaaATCATCCAGTAAACCGCC |
| cDlx1-B2-5P1 | CCTCGTAAATCCTCATCAaaTCACATAGGGGTAGCCCAGCGGTCG |
| cDlx1-B2-5P2 | GGTTGCCGGAGTGGCTGCTCACCGAaaATCATCCAGTAAACCGCC |
| cDlx1-B2-6P1 | CCTCGTAAATCCTCATCAaaTGTCGGGCTGCGAGTGGCCCGCCGA |
| cDlx1-B2-6P2 | AGAAGGACGAAGCTGTGCTGTACGCaaATCATCCAGTAAACCGCC |
| cDlx1-B2-7P1 | CCTCGTAAATCCTCATCAaaGGGACATGGGAGAAGGAGACATTTG |
| cDlx1-B2-7P2 | GTAAACAGTGCATGGAATAGTGTCCaaATCATCCAGTAAACCGCC |
| cDlx1-B2-8P1 | CCTCGTAAATCCTCATCAaaGCCTTCCCCGAGACGGGGCTGTTTA |
| cDlx1-B2-8P2 | CCGGGCGGCCCGAACTCCATAAAGAaaATCATCCAGTAAACCGCC |
| cDlx1-B2-9P1 | CCTCGTAAATCCTCATCAaaTCTCGCCGCTGGGTCTGTGTGTGTG |
| cDlx1-B2-9P2 | CTCTCAGGCATGGTGGTCATGGTCAaaATCATCCAGTAAACCGCC |
| cDlx1-B2-10P1 | CCTCGTAAATCCTCATCAaaAAAGAACCCAATTTAAGCGGAACAG |
| cDlx1-B2-10P2 | GGGGTCTGTTGTGTTTCTCAGGACGaaATCATCCAGTAAACCGCC |
| cDlx1-B2-11P1 | CCTCGTAAATCCTCATCAaaTTTCGGCTCGCGGCGGGAAAAAACG |
| cDlx1-B2-11P2 | TTGTCCGTGATAATTTGATTTTTCTaaATCATCCAGTAAACCGCC |
| cDlx1-B2-12P1 | CCTCGTAAATCCTCATCAaaGCATAGCTTCTTGGTGGGCCGAGGG |
| cDlx1-B2-12P2 | TTCGGGCTCACATAAGCTGCGGCTGaaATCATCCAGTAAACCGCC |

**Chick *Lhx8* HCR probe**

| Name | Sequences |
| --- | --- |
| cLhx8-B3-1P1 | GTCCCTGCCTCTATATCTttAGGGCGATAGAGCGGCGGAGGAAGA |
| cLhx8-B3-1P2 | CCGAGGCCATGGACCGCGGCGAAGAttCCACTCAACTTTAACCCG |
| cLhx8-B3-2P1 | GTCCCTGCCTCTATATCTttCCGAGCGCGGGCAGGTCCTTATCCT |
| cLhx8-B3-2P2 | GCGGGGCTCTCCTTCTCCGAGGTGTttCCACTCAACTTTAACCCG |
| cLhx8-B3-3P1 | GTCCCTGCCTCTATATCTttAATACATGGCCCTGCCCTACCGCAT |
| cLhx8-B3-3P2 | TGCACACGAACATCGGGTCGCTCTTttCCACTCAACTTTAACCCG |
| cLhx8-B3-4P1 | GTCCCTGCCTCTATATCTttCAACTGTCAGTCCTTTCTTTTAGGC |
| cLhx8-B3-4P2 | CTCACAAAGAATTGTAAACATTTTGttCCACTCAACTTTAACCCG |
| cLhx8-B3-5P1 | GTCCCTGCCTCTATATCTttCAATACAAAAAGGTGTTCAATAAGG |
| cLhx8-B3-5P2 | ACGTAACTAGAGTGATCTACTTCCAttCCACTCAACTTTAACCCG |
| cLhx8-B3-6P1 | GTCCCTGCCTCTATATCTttGGCTAAATGTATACTTACATATACC |
| cLhx8-B3-6P2 | ATGCAGAATCCATAGAAGTGTCTTGttCCACTCAACTTTAACCCG |
| cLhx8-B3-7P1 | GTCCCTGCCTCTATATCTttATCCTTAATAGCTTATATCCCTGAC |
| cLhx8-B3-7P2 | CATACACAGTAAGTGACTTAAGCTTttCCACTCAACTTTAACCCG |
| cLhx8-B3-8P1 | GTCCCTGCCTCTATATCTttGTTGTGTCATTGAATGGGGTAGCAA |
| cLhx8-B3-8P2 | AAAGAAATTAGGTATGACTTATTGGttCCACTCAACTTTAACCCG |
| cLhx8-B3-9P1 | GTCCCTGCCTCTATATCTttGCATCCATATAACTATGCAGGGCAG |
| cLhx8-B3-9P2 | TGGAGTCCAAGAGCTGTAGGTGAATttCCACTCAACTTTAACCCG |
| cLhx8-B3-10P1 | GTCCCTGCCTCTATATCTttTGAATATGCCATTTCTTCTAACATG |
| cLhx8-B3-10P2 | CATAGTCCCATCTTGGGGTACATATttCCACTCAACTTTAACCCG |
